# Non-Contact Measurement of Empathy Based on Micro-Movement Synchronization

**DOI:** 10.3390/s21237818

**Published:** 2021-11-24

**Authors:** Ayoung Cho, Sung Park, Hyunwoo Lee, Mincheol Whang

**Affiliations:** 1 Department of Emotion Engineering, University of Sangmyung, Seoul 03016, Korea; joa6391@gmail.com (A.C.); lhw4846@naver.com (H.L.); 2 School of Design, Savannah College of Art and Design, Savannah, GA 31401, USA; spica7601@gmail.com; 3 Department of Human Centered Artificial Intelligence, Sangmyung University, Seoul 03016, Korea

**Keywords:** video content empathy, micro-movement synchronization, non-contact empathy measurement, empathic advertisement

## Abstract

Tracking consumer empathy is one of the biggest challenges for advertisers. Although numerous studies have shown that consumers’ empathy affects purchasing, there are few quantitative and unobtrusive methods for assessing whether the viewer is sharing congruent emotions with the advertisement. This study suggested a non-contact method for measuring empathy by evaluating the synchronization of micro-movements between consumers and people within the media. Thirty participants viewed 24 advertisements classified as either empathy or non-empathy advertisements. For each viewing, we recorded the facial data and subjective empathy scores. We recorded the facial micro-movements, which reflect the ballistocardiography (BCG) motion, through the carotid artery remotely using a camera without any sensory attachment to the participant. Synchronization in cardiovascular measures (e.g., heart rate) is known to indicate higher levels of empathy. We found that through cross-entropy analysis, the more similar the micro-movements between the participant and the person in the advertisement, the higher the participant’s empathy scores for the advertisement. The study suggests that non-contact BCG methods can be utilized in cases where sensor attachment is ineffective (e.g., measuring empathy between the viewer and the media content) and can be a complementary method to subjective empathy scales.

## 1. Introduction

Empathy, a crucial factor in successful digital content marketing [1], is generally conceptualized as a multidimensional construct that includes both cognitive and affective responses to others in dyadic interactions [2,3,4]. However, empathy for digital content involves the emotional engagement of a viewer with a character in a causal and probable narrative [5]. For example, eliciting a consumer’s emotions congruent to content emotions may maximize an advertisement’s effect. Viewers empathizing with content tend to better understand the story and have more positive attitudes. They are more attentive and engaged [6,7,8], feel favorably toward products and brands [9,10], and are less likely to skip an advertisement [11,12]. Moreover, heightened empathy promotes the consumption of content in addition to attitudinal acceptance [13,14]. Such behavioral acceptance implies that viewer empathy is a critical predictor of the success of media content.

Empathy has been measured to predict the success of commercials. Escalas and Stern developed a battery of scale items to measure empathy toward advertisements, which has been widely used in consumer research [15]. Other prominent subjective measures include Schlinger’s Viewer Response Profile [16,17,18], the Balanced Emotional Empathy Scale [19], the Empathy Quotient [20], the Toronto Empathy Questionnaire [21], the Interpersonal Reactivity Index [22,23], the Basic Empathy Scale [24], and the Hogan Empathy Scale [25].

However, such subjective evaluations cannot measure the dynamics of empathy over time. Empathic questionnaires are limited to assessing dispositional empathy, which refers to an individual’s capability (i.e., personality trait) to empathize with others.

The dynamics of empathy when consuming digital content require a novel measurement that can capture the fluctuation of emotions over time. The ever-changing interplay between the viewer’s emotions and the content emotions demands a more direct, sensitive, and real-time measurement, such as physiological measures, to properly assess the degree of empathy. The unconscious level of empathy that is not verbally reportable (i.e., subjective evaluation) can be acquired through more direct physiological measures.

### 1.1. Psychophysiological Basis of Empathy

Empathy includes motor mimicry and emotional contagion associated with autonomically activated neural mechanisms of the other’s feelings [26,27,28,29]. It also includes mirroring responses between people, in which explicit and implicit physiology become synchronized [30,31,32]. Explicit responses from empathy involve the synchronization of faces, gestures, and body movements. Changes in body motion synchronization are associated with the degree of empathy during face-to-face communication [33,34]. Greater synchronization of head motion was observed when a listener empathized with a speaker in a lecture [35]. In addition, body synchronization was reported between counselors and clients when they shared empathy [36,37].

Such observable synchronized behavior is a result of an implicit empathic response. The implicit process constitutes the synchronization of physiological activities between individuals [38], which can be measured through electroencephalography (EEG) [39,40], electrocardiography (ECG) [41,42,43,44], and skin conductance [45,46]. For example, the synchronization of electrodermal activities (i.e., skin response) between a therapist and a patient correlates to the patient’s perceived empathy toward the therapist [47,48].

Neuroscientific bases have been identified for the synchronization of brain activity among participants during empathic communication [49,50,51]. Empathy researches using EEG have been mainly focused on understanding the sharing of painful experiences. Several asymmetries or activations in the pain-related brain areas have been reported, which were elicited by empathy. The left frontal asymmetry has been related to the suffering of the other, and the right frontal asymmetry has been associated with the pain and sorrow of the other [52]. Moreover, empathy-related activation in fronto-insula and anterior cingulate cortices was reported, which have been related to pain [53]. Peng et al. have shown that brain-to-brain synchronization could be triggered by sharing painful experiences and could strengthen social bonds [54].

### 1.2. Cardiovascular Measures of Empathy

Measures of cardiovascular activity reflect both attentional and affective states [55]. Cardiovascular measures can be achieved using a piezoelectric transducer, ECG, or analysis of facial micromovements. Cardiovascular activity in empathy research has been understudied compared to other physiological measures [56], but recent advances in vision technology have shed light on novel and innovative methodologies such as remote ballistocardiography (rBCG).

Kodama et al. [57] examined a psychotherapy session between a counselor and a client and found synchronization in heart rate, suggesting a promising indicator that leads to the building of rapport and empathy. Salminen et al. [58] found that higher synchrony in respiration rates, which has a positive relationship with heart rate, is associated with higher empathy. The synchronization of the heart rate can also enhance closeness [59] and intimacy [60]. 

However, the measurement of synchronization between the cardiovascular activities of viewers and people in media content has been less studied, mainly due to multiple technical issues.

First, viewers need a sensor attachment to capture physiological measurements, which is a significant barrier to general adoption. Second, to evaluate empathy, measuring dyadic synchronization is paramount. The cardiovascular information of both the viewer and the person in the media content must be obtained and analyzed. Obviously, acquiring the latter is impossible with sensor attachment because it is digital content.

However, advances in vision technology for cardiac measurements, such as remote photoplethysmography (rPPG) and rBCG, suggest promising methods for overcoming these challenges. The rPPG evolved to detect changes in blood volume remotely without direct contact between the photosensor (i.e., PPG) and the skin [61]. Non-contact data acquisition is possible through various means, including infrared [62], thermal [63], and RGB [64] cameras. The rPPG uses band-pass filters to eliminate motion components in images [65] but has less effect on cardiovascular activities that include the motion itself, referred to as ballistocardiography motion [66]. The rBCG is a measurement of ballistocardiographic head movements through remote means using a camera and vision-based analysis. These vision technologies have improved considerably in recent years, enabling the estimation of the heartbeat signals of both the viewer and the person in the digital content without needing skin contact.

Specifically, BCG motion causes microscopic vibration (i.e., micro-movement), which appears in the face through the carotid artery [67]. Micro-movement implies the subtle movement of a face that the human eye cannot easily see. This is caused by regular vibrations from the heart that are transmitted to the face. Micro-movement can be obtained by filtering the frequency corresponding to the regular heart rate band from the frontal facial video capture [68,69,70,71]. Analyzing the similarity of micro-movement between viewers and digital content (e.g., advertisements) may provide insights into whether the viewer is empathizing with the content. We intended to analyze the similarity of micro-movements through cross-entropy analysis and compare it to the participants’ subjective empathy through a questionnaire. To our knowledge, no study has investigated the relationship of micro-movements through an rBCG method for a participant and a person in real-world media content, such as an advertisement.

## 2. Materials and Methods

### 2.1. Research Hypothesis

This study sought to verify the following hypothesis:

**Hypothesis** **1** **(H1).** *The more similar the micro-movements between the participant and the person in the advertisement, the higher the participant’s empathy scores for the advertisement.*

The following section explains our operational definition of micro-movement signals, how the signals were measured from the participant and the advertisement, and how the participant’s subjective assessment of empathy was acquired.

### 2.2. Experimental Design

The main experiment was a one-factor design (empathy factor) with two levels (empathy and non-empathy). Each participant viewed two empathy conditions (i.e., within-subject design), manifested in an empathy or non-empathy advertisement, and responded to an empathy questionnaire. The design of the stimuli (i.e., advertisement) and the questionnaire are explained in Section 2.3.

The dependent measurements involved the similarity of micro-movements between the participant and the stimulus, specifically, the similarity between the micro-movement signals extracted from the participant and those from the person in the advertisement. Cross-entropy was used as a similarity metric. Cross-entropy is suitable for the comparison of periodic distributions. The more similar the two distributions, the closer the cross-entropy is to zero [72]. This study extracted the micro-movement signals by filtering the power spectrum between 0.75 Hz and 2.5 Hz corresponding to 45~150 bpm when static. However, this filtering range may vary according to the context, situation, and use cases. The details of the analysis are explained in Section 3.

### 2.3. Participants

Thirty participants (15 males and 15 females) voluntarily participated in the experiment. The mean age of participants was 22 (±2) years. None of the participants had a medical history of cardiovascular disease. The participants had an uncorrected or corrected visual acuity of 0.6 or better and were able to wear soft contact lenses but not glasses. Written informed consent was obtained from all the participants prior to the experiment. All participants were compensated for their participation.

Empathy varies with demographic characteristics, such as age [28], race [73], education [74], and gender [75]. Researchers have suggested an inverse-U-shaped pattern as a function of age, with middle-aged adults showing higher empathy than young adults [28]. Meta-analyses of gender differences in empathy support that women have more empathy than men [28,75,76]. One study reported a decline in empathy among undergraduate nursing students as they advanced through training [74]. The empathic neural response is increased for members of the same race, but not for other races [73]. Due to such demographic variance, the most recent (2021) massive survey (*n* = 3486) on the experience of empathy [77] quota sampled to reflect the U.S. population on demographic parameters. However, all empirical lab studies on empathy, including ours, have limitations when generalizing. We balanced the N of gender (15) and confirmed that gender did not have an effect on the dependent measures and ensured that the ethnicity of the participants (i.e., Korean) was consistent with the characters in the video stimuli. However, we acknowledge the limitation for generalizing the findings, such that the results may only apply to younger adults. Further studies are needed to confirm this hypothesis.

### 2.4. Procedures and Materials

**Figure 1:**
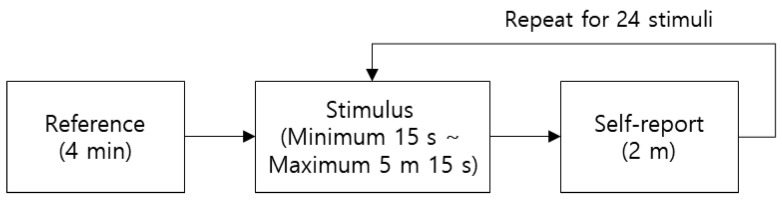
Experimental procedure.

The experimental procedure is shown in Figure 1. The participants stared at the blank screen for four minutes to stabilize their physiological state. For each stimulus, participants viewed an advertisement video and responded to a self-report questionnaire. Each condition (empathy and non-empathy) had 12 stimuli, so participants viewed 24 advertisements in total. The stimuli were presented in random order.

**Figure 2:**
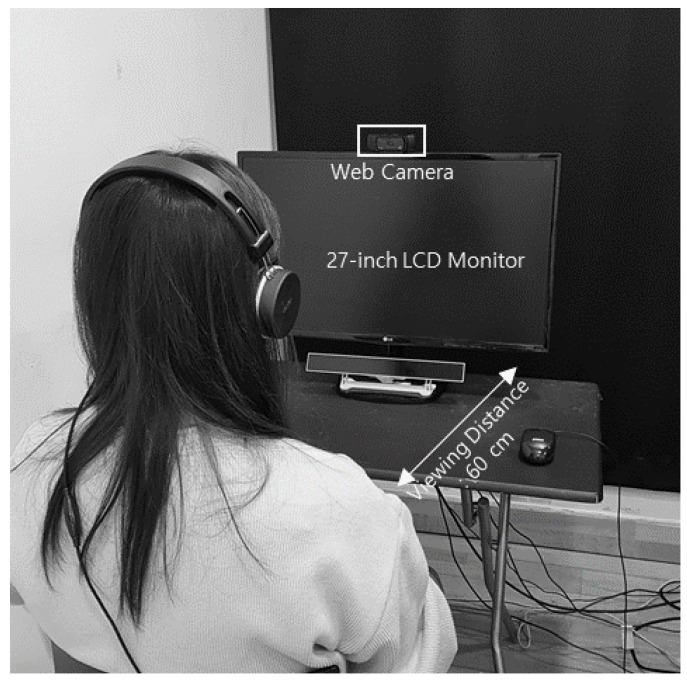
Experimental environment.

Participants’ frontal views, which were necessary for extracting the micro-movement signals, were recorded at 30 fps, 1920 × 1080 pixels, using a web camera installed on the monitor while they viewed the stimuli, as shown in Figure 2.

#### 2.4.1. Video Stimuli (Advertisements)

Marketing researchers have explored empathy as a construct for estimating advertising effects. Escalas and Stern suggested that well-developed stories elicit higher levels of empathy than poorly developed ones [15]. Classical drama advertisements that have clear causality have been better able to hook viewers into commercials than vignettes. Emotionally driven advertisements have a positive impact on consumers’ engagement and empathy [8,78,79]. In short, advertisements that elicit viewers’ empathy tend to provide a clear context behind the story, in addition to an emotional appeal [14,79,80]. As a result, we chose three criteria for selecting the video stimuli: (1) causality of the storyline, (2) advertising appeal type, and (3) the degree of empathy.

Nine emotion researchers viewed and assessed 50 candidate advertisements. The candidates were limited to those targeting the younger generation in their 20 s and 30 s, consistent with the participant pool. For each criterion related to the candidate, the researchers responded from −3 to +3 on a six-point Likert scale. Per criteria 1, researchers scored from −3 (ambiguous causality) to +3 (clear causality) for the story of the advertisement. Per criteria 2, they scored from −3 (rational appeal) to +3 (emotional appeal) for the advertising appeal type. Finally, according to criteria 3, they scored from −3 (not empathetic) to +3 (empathetic).

We classified the candidates into empathy advertisements if the average score for the evaluators was above zero for all three criteria. Conversely, we classified them into non-empathy advertisements if the score was below zero. For each advertisement group (empathy and non-empathy), we sorted the advertisements into four product advertisements (energy boosters, snacks, computer peripheral devices (e.g., printer)) and selected the three best advertisements for each product group. That is, we selected 12 advertisements for each condition (empathy and non-empathy).

Empathy advertisements tend to be longer than non-empathy advertisements because the viewer requires some time for the narrative to “sink in”. In contrast, non-empathy advertisements focus on the presentation of prominent models and products. For example, an energy booster’s empathy advertisement has a story involving a student exhausted from studying being revitalized after drinking an energy drink. The non-empathy advertisement, however, featured a character dancing with an energy drink and did not have a particular narrative.

#### 2.4.2. Subjective Evaluations

As empathy is a multifaceted construct that includes both cognitive and affective processes, we adopted a comprehensive and empirically validated questionnaire with the participants’ ethnicity (i.e., Korean). We used the Consumer Empathic Response to Advertising Scale [81,82], which consists of 11 items, as shown in Table 1. The factor loading exceeded 0.4 and Cronbach’s alpha exceeded 0.8. The questionnaire included three empathy factors: cognitive empathy, affective empathy, and identification empathy. The dependent variable for analysis was the sum of all 11 items.

All questions were rated on a seven-point Likert scale. We asked for the degree of agreement with each empathy statement, with the lowest scale labeled “strongly disagree” and the highest scale labeled “strongly agree”. The survey was collected through a web survey rather than a paper questionnaire.

## 3. Analysis

This study aimed to analyze whether the similarity of micro-movement signals between participants and advertisements differs according to the user’s perceived empathy (i.e., subjective evaluation) with the advertisement video. The signal processing to filter only the micro-movements caused by the heartbeat is described in detail in Section 3.1. In addition, a method for calculating the cross-entropy, an indicator of similarity between the two signals, is described. Section 3.2 describes the statistical difference in the similarity between the participant and advertisement measured by cross-entropy according to the empathy score.

### 3.1. Signal Processing

**Figure 3:**
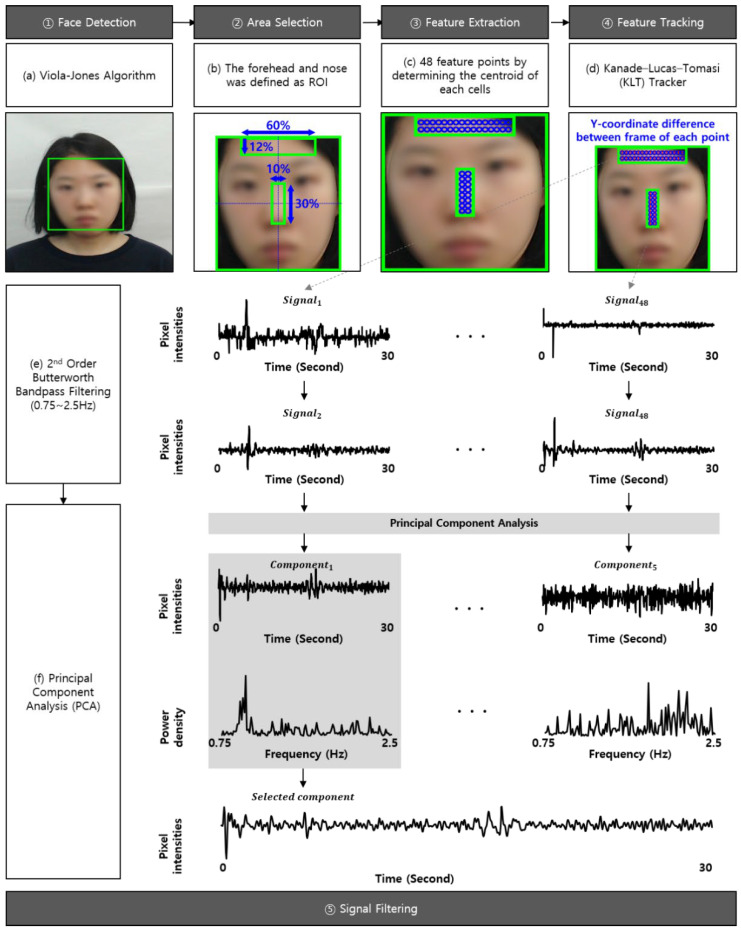
Signal processing of the micro-movements [71]. (**a**) Face detection using Viola-Jones algorithm; (**b**) Area selection using the forehead and nose defined as ROIs; (**c**) Feature extraction using the GFTT algorithm; (**d**) Feature tracking using the KLT tracker; (**e**) Bandpass filtering for signals in 30 s window buffer using the second order Butterworth filter; (**f**) Decomposition of noise using PCA.

The micro-movement signals were measured from the participant’s facial videos, as shown in Figure 3. Ballistocardiographic changes are reflected to the face and can be measured at a distance, as validated by Balakrishnan [68]. The face was detected from the facial video using the Viola-Jones face detector and was defined as a region of interest (ROI). As the forehead and nose were more robust to facial expressions than other facial regions, the ROI was divided into multiple ROIs by cropping to the middle 60% of the width and top 12% of the height (i.e., forehead region) and the middle 10% of the width and middle 30% of the height (i.e., nose region).

Determining the feature point within multiple ROIs was necessary to measure the movements induced by the BCG. Although several studies on remote BCG employed the good-feature-to-track (GFTT) algorithm [83,84], their feature point numbers were not fixed because the algorithm determined the feature points based on the solid edge components. It was difficult to employ the GFTT algorithm in this study because the feature points needed to be re-determined quickly owing to the frequent change of the screen and the face movement.

Thus, the ROIs of the forehead and nose regions were divided into cells using 16 × 2 and 2 × 8 grids, respectively. This study employed 48 feature points by determining the centroid of each cell as a feature point. The movements were measured by tracking the y-coordinate difference between frames of each feature point using the Kanade-Lucas-Tomasi (KLT) tracker because the BCG movements were generated up and down by the heartbeat [85,86,87].

The movements measured from the face are a combination of facial expressions, voluntary head movements, and micro-movements. Therefore, it is essential to remove motion artifacts due to facial expressions and voluntary head movements from the measured movements. First, the movements were filtered by a second order Butterworth bandpass filter with a cut-off 0.75–2.5 Hz corresponding to 45–150 bpm. Then, the movements were normalized from their mean value (i.e., *μ*) and standard deviation (*σ*) by z-score. If the movements exceeded the *μ* + −2*σ*, they were determined to be noise, due to the subtle movements, and their mean value (i.e., *μ*) was corrected. Finally, principal component analysis (PCA) was performed to estimate the micro-movement from the mixed movements by decomposing the noise from facial expressions and voluntary head movements. This study extracted five components using PCA and then selected one component with the highest peak in their power spectrum converted using a fast Fourier transform. The selected component was finally determined to be micro-movements.

### 3.2. Statistical Analysis

As empathy is an individualized experience, the manner in which each stimulus affects each participant varies. Individualized response is affected by factors, such as the individual’s empathy capability, predisposed tendency, and past experience (for an extensive review of empathy as a concept, see [88]). The observer’s (i.e., the person who empathizes) mood and personality are also an important modulating factor [89]. Such individual differences mean that, in our study, the empathy stimuli selected by the emotion experts do not necessarily elicit empathy from the participants. Therefore, we applied an inclusion criterion to the participants’ subjective empathy scores to select response sets from certain stimuli for analysis. We selected data obtained from stimuli that scored, on average (i.e., the mean of all 30 participants), on or higher than four for the empathy condition. In the seven-point Likert scale, four was the middle point, labeled as “Neutral”. Conversely, we selected data obtained from stimuli that scored less than four on average for the non-empathy condition. This selection process yielded response sets from four out of the original 12 stimuli in the empathy condition and six out of the original 12 stimuli in the non-empathy condition.

In short, we analyzed 60 samples (30 participants in two empathy conditions) consisting of subjective empathy scores and cross-entropy data. A paired *t*-test was used to test this hypothesis.

## 4. Results

**Figure 4:**
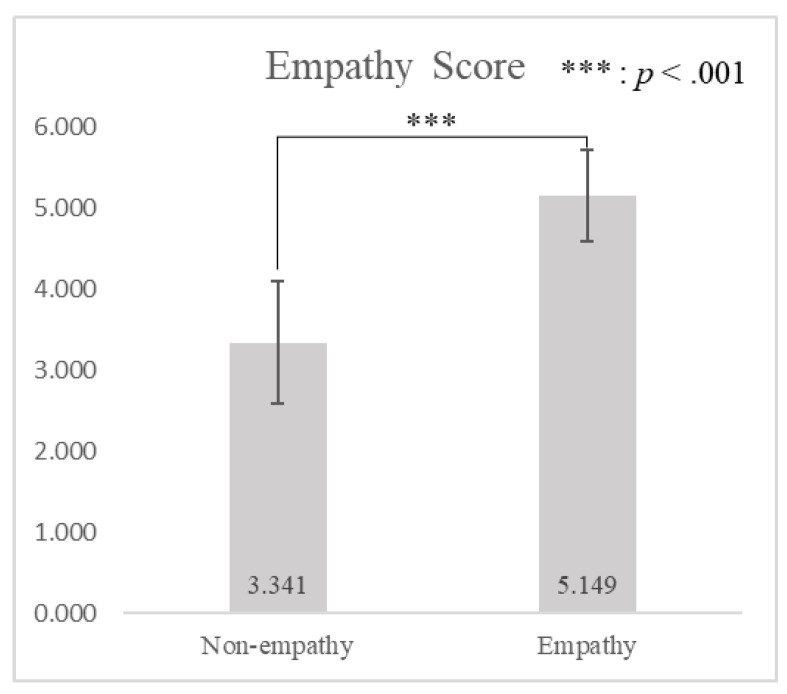
A comparison of empathy scores for non-empathy and empathy advertisements by paired *t*-test.

The study analyzed differences in the micro-movement similarity between empathy and non-empathy conditions using a *t*-test. The results showed that there was a significant difference in the subjective empathy score between empathy and non-empathy conditions induced by advertisements (*t*(29) = −11.754, *p* < 0.001), as shown in Figure 4. The subjective empathy score was significantly higher when watching empathy advertisements (*μ* = 5.149, *σ* = 0.564) than non-empathy advertisements (*μ* = 3.341, *σ* = 0.759).

**Figure 5:**
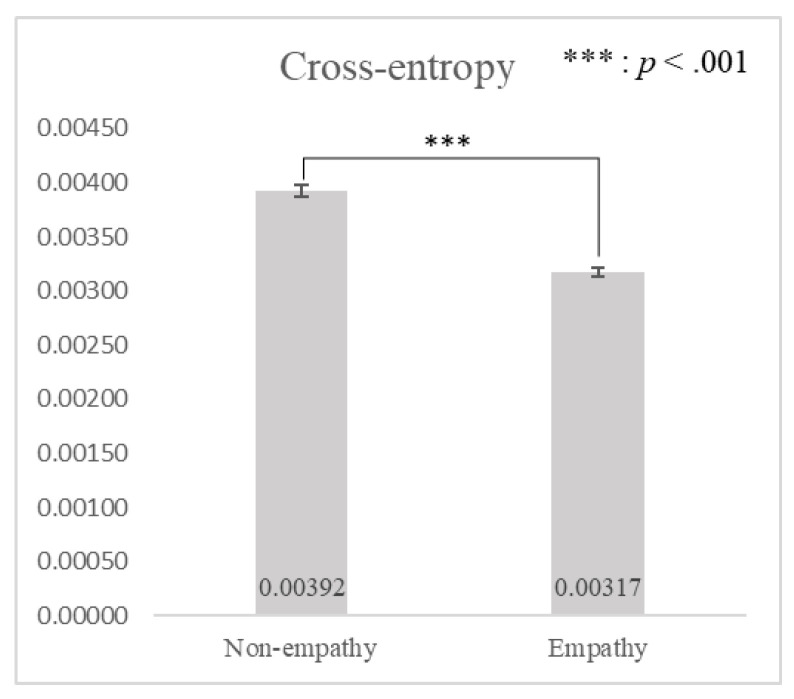
A comparison of cross-entropy between non-empathy and empathy advertisements by paired *t*-test.

There was a significant statistical difference in cross-entropy between empathy and non-empathy advertisements (*t*(29) = 61.019, *p* < 0.001), as shown in Figure 5. As predicted, cross-entropy was significantly lower when watching empathy advertisements (*μ* = 0.00317, *σ* = 0.00005) than non-empathy advertisements (*μ* = 0.00392, *σ* = 0.00005). This supported hypothesis *H*_1_, which stated that the more similar the micro-movements (i.e., the lower the cross-entropy) between the participant and person in the advertisement, the higher the participant’s empathy scores for the advertisement (i.e., empathy advertisements).

The Pearson correlation indicated that cross-entropy was also significantly associated with empathy score (*r* = −0.796, *p* < 0.001), indicating an inverse relationship between cross-entropy and the empathy scores. That is, the lesser cross-entropy, the higher the empathy scores.

## 5. Discussion

In summary, our study invited participants to view advertisements classified as empathy or non-empathy advertisements by experts. During each viewing of the advertisement, we recorded their facial data and obtained their subjective empathy scores after each viewing. We analyzed the cross-entropy between the participant’s and the person’s facial data and found that it was significantly lower when viewing empathy advertisements than when viewing non-empathy advertisements.

To the best of our knowledge, this is the first study to apply remote BCG methods to understand empathy-based micro-movement synchronization in a real-world use case (i.e., viewing an advertisement). Our research confirmed that the higher the similarity of micro-movement between the participants and the advertisements, the higher the subjective empathy. The results validate the remote BCG methods with the accompanying analysis process (e.g., cross-entropy analysis), suggesting an alternative or complementary method to the subjective empathy scales.

Our findings also provide implications for understanding the empathic interactions of human dyads. In human communication, information is shared through natural language (i.e., explicit channels), whereas empathy is mainly shared through embodied synchrony (i.e., implicit channels). The latter synchronization is widely observed in human communication and is reflected in the harmonization of the heart rhythm. In other words, the heartbeat tends to follow the rhythm of someone who empathizes. Such mutual entrainment has been defined as two interacting nonlinear oscillating systems with different periods becoming a common period [90]. Although challenging, advances in technology enable us to tap into heartbeat traces through the carotid artery, reflected in the facial micro-movement. Our study confirmed that microscopic vibration is a valid indicator of dyadic empathy synchronization in an ecologically valid scenario.

In previous studies that measured empathy based on unconscious physiological responses [41,43], it was also verified that the correlation between the heartbeat patterns of two people was higher in the empathy condition than in the non-empathy condition. They measured heart rate patterns by attaching an ECG sensor to the participant’s skin. The task of eliciting empathy was overly simplified, such as facing each other, and only momentary emotions were of concern, resulting in limitations to generalization. Although they can effectively elicit a definite empathic response, the emotion dynamics were not considered. 

In addition, there were fewer applications measuring empathy for digital content because of the challenge in solving the barrier of obtrusive measurement and consideration of the dynamic nature of empathy. This study suggested a practical method for measuring empathy that complements the issue of contact-based empathy measurement that obstructs users’ immersion in the content.

The hypothesis of the present study was tested under experimental conditions by manipulating product advertisements. This study acknowledges that there were large differences among the durations of the stimuli, and the stimuli were only focused on product commercials. However, the differences in time duration among stimuli did not affect the similarity because the similarity between the two signals was analyzed in the frequency domain. That is, because the similarity of the periodicity of the two signals was analyzed, the time length of the signal did not have a significant effect. Even if there was an effect, the empathy stimuli, which had a long duration, were difficult to make similar to the non-empathy stimuli, which had a short duration, because they had to vibrate at a similar frequency for a longer period of time.

This study suggests an application framework for evaluating empathy in interaction (e.g., viewing) with digital content. As our suggested method is non-contact and unobtrusive to real-life behavior (e.g., consuming media), future research agendas seem promising. Specifically, future studies may investigate content in other media domains (e.g., movies, TV shows, video games).

However, we acknowledge that a larger N would be needed to achieve the appropriate power to completely rule out false positives. We acknowledge that our N is small (30) and, as such, we conducted a post hoc power analysis with the program G*Power [91] with power set at 0.8 and α = 0.05, d = 0.5, two-tailed. The results suggest that an N value of approximately 34 would be needed to achieve appropriate statistical power.

Empathy is a multifaceted social psychological construct that is affected by many factors, such as the relationship and history between the observer (i.e., empathizer) and the observed. Such social relationships are also shaped by intimacy, while favorability also comes into play. As empathy is dependent on context and task [89,92], our study has an inherent limitation in generalization.

We also acknowledge that empathic expression is a result of a combination of many nonverbal modalities (e.g., voice, facial expression, posture). We focused on a singular modality, the facial movements captured from the involuntary heartbeat, because such measures could also be confounded by noise. Moreover, there can be a gap between the actual emotion the actor felt and the physiological measurement we acquired. Such a gap can be measured through a combination of expressive measures (facial muscle movement, gestures) and implicit measures (heart rate, GSR). Future studies may investigate multimodal recognition of empathy, in addition to facial micro-movements.

We strived to filter out the signals that represent empathy from the signal spectrum as closely as possible to the target population by guiding the participant not to move and to refrain from exaggerating facial expressions. We did not include any participants who may have made significant movements that would confound our results, such as participants with Tourette syndrome or a person with bruxism. 

Privacy issues that may arise from identifying individuals can be crucial in research that considers prosocial behaviors. However, the suggested method of recognizing empathy can enhance privacy by not saving personal identification data (i.e., original record video) in the database. Only the processed secondary data (i.e., micro-movement signals) can be saved in the database by analyzing video frames in real-time without recording the face images. Then, the synchronization data can be analyzed if only a key can match (i.e., random number) an advertisement and a viewer. The analyzed micro-movement features are hardly restored to the original facial image, so it is impossible to identify its data.

## Figures and Tables

**Table 1:** Questionnaire about Empathy to Video Contents.

	Questionnaire	Empathy Factor
1	I understood the characters’ needs.	Cognitive empathy
2	I understood how the characters were feeling.	
3	I understood the situation of the video.	
4	I understood the motives behind the characters’ behavior.	
5	I felt as if the events in the video were happening to me.	Affective empathy
6	I felt as if I was in the middle of the situation.	
7	I felt as if I was one of the characters.	
8	I experienced many of the same feelings that the characters portrayed.	Identification empathy
9	I felt the characters’ needs were similar to mine.	
10	The events in the video were similar to my experience.	
11	I felt as if the events in the video could happen to me.	
